# Mutation in the CCAL1 locus accounts for bidirectional process of human subchondral bone turnover and cartilage mineralization

**DOI:** 10.1093/rheumatology/keac232

**Published:** 2022-04-12

**Authors:** Alejandro Rodríguez Ruiz, Marcella van Hoolwerff, Sara Sprangers, Eka Suchiman, Ton Schoenmaker, Petra Dibbets-Schneider, Johan L Bloem, Rob G H H Nelissen, Christian Freund, Christine Mummery, Vincent Everts, Teun J de Vries, Yolande F M Ramos, Ingrid Meulenbelt

**Affiliations:** Department of Biomedical Data Sciences, Section Molecular Epidemiology, Leiden University Medical Center, Leiden; Department of Biomedical Data Sciences, Section Molecular Epidemiology, Leiden University Medical Center, Leiden; Department of Oral Cell Biology; Department of Biomedical Data Sciences, Section Molecular Epidemiology, Leiden University Medical Center, Leiden; Department of Oral Cell Biology; Department of Periodontology, Academic Centre for Dentistry Amsterdam (ACTA), University of Amsterdam and Vrije Universiteit , Amsterdam; Department of Radiology; Department of Radiology; Department of Orthopedics, Leiden University Medical Center, Leiden, The Netherlands; Department of Anatomy; LUMC hiPSC Hotel; Department of Anatomy; LUMC hiPSC Hotel; Department of Oral Cell Biology; Department of Oral Cell Biology; Department of Periodontology, Academic Centre for Dentistry Amsterdam (ACTA), University of Amsterdam and Vrije Universiteit , Amsterdam; Department of Biomedical Data Sciences, Section Molecular Epidemiology, Leiden University Medical Center, Leiden; Department of Biomedical Data Sciences, Section Molecular Epidemiology, Leiden University Medical Center, Leiden

**Keywords:** hiPSCs, CRISPR/Cas9, *TNFRSF11B*, osteomorph, matrix mineralization

## Abstract

**Objectives:**

To study the mechanism by which the readthrough mutation in *TNFRSF11B*, encoding osteoprotegerin (OPG) with additional 19 amino acids at its C-terminus (OPG-XL), causes the characteristic bidirectional phenotype of subchondral bone turnover accompanied by cartilage mineralization in chondrocalcinosis patients.

**Methods:**

OPG-XL was studied by human induced pluripotent stem cells expressing OPG-XL and two isogenic CRISPR/Cas9-corrected controls in cartilage and bone organoids. Osteoclastogenesis was studied with monocytes from OPG-XL carriers and matched healthy controls followed by gene expression characterization. Dual energy X-ray absorptiometry scans and MRI analyses were used to characterize the phenotype of carriers and non-carriers of the mutation.

**Results:**

Human OPG-XL carriers relative to sex- and age-matched controls showed, after an initial delay, large active osteoclasts with high number of nuclei. By employing hiPSCs expressing OPG-XL and isogenic CRISPR/Cas9-corrected controls to established cartilage and bone organoids, we demonstrated that expression of OPG-XL resulted in excessive fibrosis in cartilage and high mineralization in bone accompanied by marked downregulation of *MGP*, encoding matrix Gla protein, and upregulation of *DIO2*, encoding type 2 deiodinase, gene expression, respectively.

**Conclusions:**

The readthrough mutation at CCAL1 locus in *TNFRSF11B* identifies an unknown role for OPG-XL in subchondral bone turnover and cartilage mineralization in humans via DIO2 and MGP functions. Previously, OPG-XL was shown to affect binding between RANKL and heparan sulphate (HS) resulting in loss of immobilized OPG-XL. Therefore, effects may be triggered by deficiency in the immobilization of OPG-XL Since the characteristic bidirectional pathophysiology of articular cartilage calcification accompanied by low subchondral bone mineralization is also a hallmark of OA pathophysiology, our results are likely extrapolated to common arthropathies.

Rheumatology key messages

- OPG-XL mutation directly affects chondrocyte and osteoblast states towards matrix mineralization mediated by respectively *MGP* and *DIO2*.
- Expression of OPG-XL drives accumulation of large active osteoclasts with high number of nuclei.
- Interference with OPG–RANKL–heparan sulphate underlying concurrent cartilage calcification and subchondral bone loss likely extrapolates to common arthropathies.

## Introduction

Joint tissue degeneration during OA is a complex multistep process characterized by a pathogenic bidirectional process of subchondral bone turnover and cartilage mineralization [1, 2]. It has been suggested that this characteristic inverse mineralization process has shared mechanisms with the frequently observed concurrent pathogenic bone turnover and vasculature mineralization [2, 3]. Key proteins likely involved are osteoprotegerin (OPG), a decoy receptor of osteoclastogenesis [4], and matrix Gla protein (MGP), a vitamin K-dependent inhibitor of ectopic bone formation [5], since overexpression or knockdown of their genes in murine models results in such an inverse pathological mineralization process [6–8]. The inverse causal role of dysfunctional OPG in human joint tissue mineralization was demonstrated by identification of a readthrough mutation at the chondrocalcinosis locus 1 (CCAL1; c1205A=T; p. Stop402Leu) [9]. The mutation in *TNFRSF11B*, encoding OPG was identified in multiple families worldwide [10–12]. In these families, the CCAL1 phenotype is defined by early onset OA with different levels of articular cartilage calcification, i.e. chondrocalcinosis [13] and low subchondral bone mineralization [12].

OPG is a well-known soluble decoy receptor that competes with RANK expressed in osteoclasts for binding to RANK ligand (RANKL) [14]. Binding of RANKL to RANK drives osteoclastogenesis and hence bone turnover, while binding to OPG inhibits this process [15]. Pleiotropic functions of OPG and RANKL were more recently suggested by showing that RANKL stimulates osteoclast fission to produce transcriptionally distinct osteomorphs, which in turn recycle towards large multinucleated osteoclasts or polykaryons by fusion under tight control of OPG [15]. Although binding of OPG to RANKL, established by N-terminal domains of OPG, is frequently studied, less is known about the interaction of OPG via its C-terminus with membrane bound heparan sulphate (HS) on osteoblasts [16]. This binding appears indispensable for RANKL-mediated inhibition of osteoclastogenesis due to immobilization of secreted OPG on the osteoblast membrane and formation of a stable HS–OPG–RANKL complex [16, 17]. In line with this, the CCAL1 readthrough mutation, adding an additional 19 amino acids to the C-terminus of OPG, denoted OPG-XL, has been shown to hamper OPG-HS binding hence permitting osteoclastogenesis and bone turnover [11]. This explains characteristic low subchondral bone density in affected CCAL1 family members [12] and sporadic cases [18].

The mechanism by which OPG-XL results in cartilage calcification remains, however, elusive. In fact, a robust role of *TNFRSF11B* and *TNFSF11*, encoding RANKL, particularly in cartilage (patho)physiology, has been highlighted by transcriptome-wide studies. Herein, *TNFRSF11B* and *TNFSF11*, but not *TNFRSF11A* encoding RANK, show high expression and are robustly responsive to OA cartilage pathophysiology as marked by consistent high upregulation in human OA affected relative to preserved [19–21] or healthy [22] cartilage. In contrast, differential expression of *TNFRSF11B* or *TNFSF11* in subchondral bone underlying preserved and lesioned areas of OA cartilage was not observed [23]. Other than that, with *TNFSF11* being a robust OA risk gene identified in the largest genome-wide association study to date [24], aberrant function of OPG/RANKL also underlies common OA aetiopathology.

Here, we set out to functionally characterize the effects of OPG-XL in joint tissues by employing mutated and control human primary chondrocytes, as well as human induced pluripotent stem cells (hiPSCs) from affected CCAL1 family members and CRISPR/Cas9 repaired hiPSC isogenic controls, to established *in vitro* organoid models of cartilage and bone [25]. Additionally, to study the effect of OPG-XL in human osteoclastogenesis, monocytes isolated from blood of carriers of the mutation were compared with monocytes of age- and sex-matched controls in osteoclastogenesis assays. Altogether, we aimed to decipher effects of OPG-XL on joint tissue mineralization that could explain the CCAL1 phenotype of articular cartilage calcification in concurrence with low subchondral bone mineralization. Given that subchondral bone turnover and cartilage calcification are general hallmarks of OA pathophysiology and at the molecular level involve *TNFRSF11B* and *TNFSF11*, our results are likely of relevance to common OA.

## Methods

### Study participants

Within the Familial early-onset OsteoArthritis (FOA) study, 13 family members were enrolled (six females and seven males aged 23–62 years with mean age 47 years; Supplementary Table S1, available at *Rheumatology* online). Cartilage samples were collected within the ongoing Research Arthritis and Articular Cartilage (RAAK) study from five common OA patients and one family member undergoing total joint replacement surgery (RAAK: two females and three males aged 50–87 years with mean age 73 years; FOA: female aged 61).

The Medical Ethics Committee of the Leiden University Medical Center (LUMC) gave approval for the RAAK study (P08.239 and P19.013), the familial OA (FOA) study (P12-256), and for generation of hiPSCs from skin fibroblasts of healthy donors (P13.080). Informed consent was obtained from all participants and donors in our manuscript.

### Genotyping and radiographic analyses

**Fig. 1:**
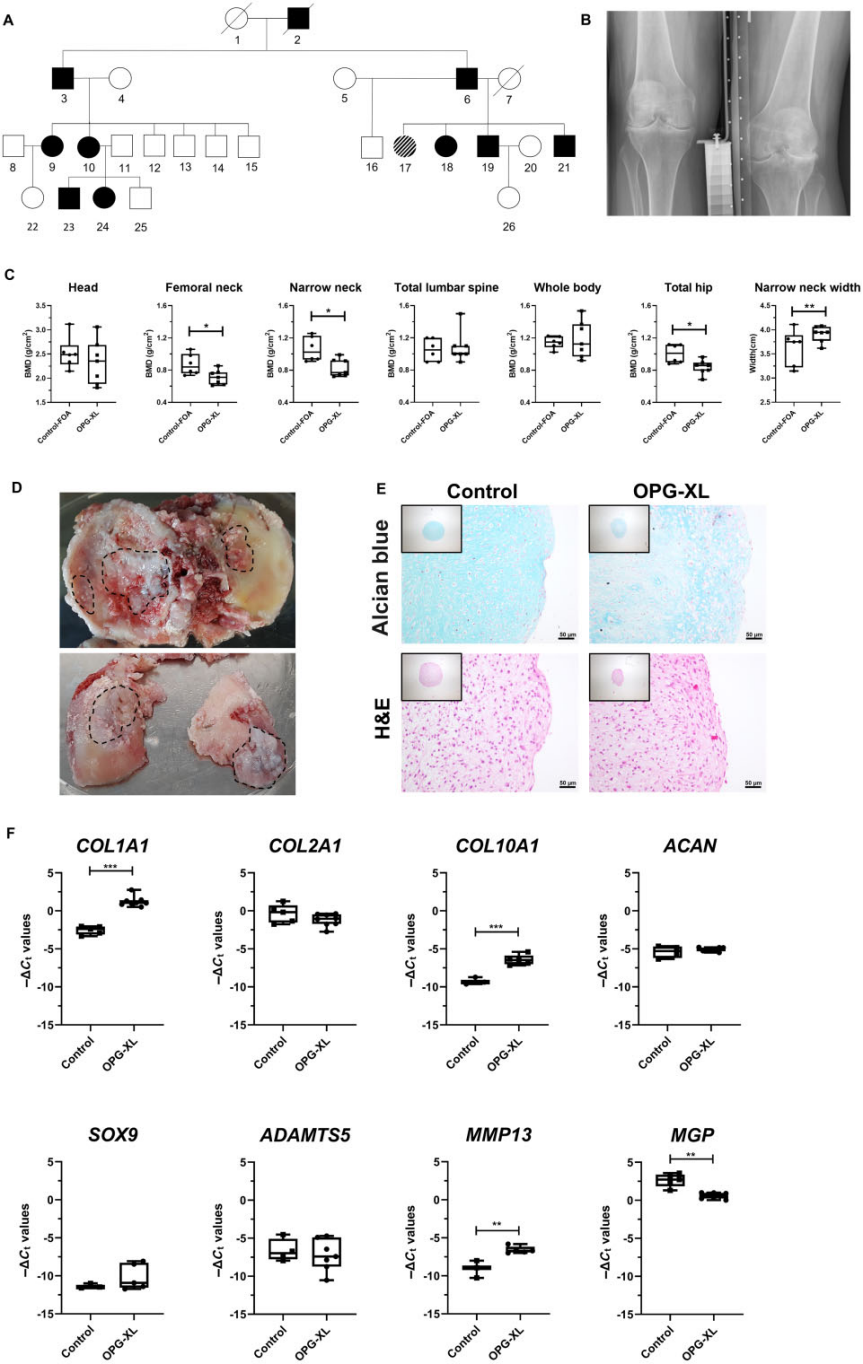
Characterization of early-onset osteoarthritis family (**A**) Pedigree of early-onset osteoarthritis family (FOA) with the *TNFRSF11B* readthrough mutation (OPG-XL). Squares represent males and circles females (black symbols represent affected individuals; diagonal lines indicate deceased family members). (**B**) Knee radiograph of individual no. 9 from the family tree, with severe articular surface destruction. (**C**) BMD of family members with and without the OPG-XL mutation as determined by dual energy X-ray absorptiometry scans and corrected for sex, age and BMI (*N*= 7 OPG-XL family members, *N* = 6 healthy family members). (**D**) Knee joint from patient that underwent replacement surgery (indicated with dashed line regions with severely calcified cartilage). (**E**) Alcian blue and H&E staining of neo-cartilage organoids derived from primary chondrocytes of common OA patients and of OPG-XL carrier (day 21 of chondrogenesis; scale bars: 50 µm). (**F**) Boxplots for −Δ*C*_t_ values of matrix genes in neo-cartilage of common OA patients (*N* = 5 patients, *n* = 1) and of OPG-XL carrier (*N* = 1 FOA, *n* = 8). *P*-values determined with generalized estimation equation while including every independent gene as dependent variable, BMD as dependent variable, and age, sex, BMI and mutation status as covariate (*P < 0.05; **P < 10^−4^; ***P < 10^−6^). *ACAN*: aggrecan; *ADAMTS5*: a disintegrin and metalloproteinase with thrombospondin motifs; H&E: haematoxylin and eosin; *MGP*: matrix Gla protein; *MMP13*: matrix metallopeptidase 13; OPG-XL: C-terminal extended osteoprotegerin encoded by *TNFRSF11B* readthrough mutation; *SOX9*: SRY-box transcription factor 9.

FOA family members were genotyped with an in-house genetic test developed by the Department of Clinical Genetics to determine presence or absence of a previously identified readthrough mutation in *TNFRSF11B* (OPG-XL; Fig. 1A) [9], and were characterized by dual energy X-ray absorptiometry (DEXA) as well as radiographs and MRI of the knees to respectively determine BMD and OA severity (Supplementary Tables S2 and S3, available at *Rheumatology* online; Fig. 1B). Further details are described in Supplementary Materials and methods, available at *Rheumatology* online.

### Generation, characterization and CRISPR/Cas9 correction of OPG-XL patient hiPSCs

Human iPSCs were generated by the LUMC iPSC core facility as described before [26] from skin fibroblasts of a FOA participant, carrier of the mutation resulting in expression of OPG-XL (line LUMC0103iOPG). Pluripotency and spontaneous differentiation were assessed, and cells were karyotyped after 15 passages (Supplementary Fig. S1A–C, available at *Rheumatology* online). Cells were maintained under standard conditions (37°C, 5% CO_2_) in TeSR-E8 medium (STEMCELL Technologies, Cologne, Germany).

To obtain two independent isogenic hiPSC controls without the mutation (lines B89 and C81), CRISPR/Cas9 correction of the mutation was performed for the OPG-XL hiPSCs (LUMC0103iOPG). For this, two single guide RNAs (sgRNAs), gRNA1 (5′-AAAAATAAGCTGCTTATTACTGG-3′) and gRNA2 (5′-AAGCTGCTTATTACTGGAAATGG-3′), were cloned into a CRISPR/Cas9 plasmid (PX458), and co-transfected using Lipofectamine Stem Reagent (Thermo Fisher Scientific, Landsmeer, The Netherlands) with a single-stranded oligo donor repair template (ssODN 5′-CACTGAAAGCCTCAAGTGC CTGAGAAACAGTTTACTCATCCATGGGATCTCGCCAATTGTGAGGAAACAGCTCAATGGCGATTTCGAGTTATAAGCAGCTTATTTTTACTGATTGGACCTGGTTACC-3′) to achieve homologous directed repair. Further details are described in Supplementary Materials and methods and Supplementary Fig. S1D–F, available at *Rheumatology* online.

### Human primary chondrocytes

Human primary chondrocytes were collected from OA patients (RAAK study; *N* = 5 donors) and carrier of OPG-XL (FOA study; *N* = 1 donor) undergoing joint replacement surgery. Collection, expansion and deposition of cartilage extracellular matrix of primary chondrocytes has been previously described [20].

### Chondrogenesis and osteogenesis of iPSCs

Mesenchymal stromal cells were generated from hiPSC (hiMSCs) of the OPG-XL hiPSCs (LUMC0103iOPG) and of the two thereof derived CRISPR/Cas9 isogenic control hiPSCs (B89 and C81) using Stemcell Technologies’ Mesenchymal Progenitor Kit following the manufacturer’s instructions. Chondrogenesis and osteogenesis was performed in organoids following our established protocol employing 750 000 cells per organoid as described before [25].

### Isolation of blood cells and osteoclastogenesis

Peripheral blood mononuclear cells (PBMCs) were isolated from whole blood of six FOA members with mutation and six sex- and age-matched controls (characteristics of donors in Supplementary Table S4, available at *Rheumatology* online) using Ficoll density gradient centrifugation as previously described [27]. Further details of osteoclastogenesis with CD14 positive monocytes are described in Supplementary Materials and methods, available at *Rheumatology* online.

### CTX-1 measurement and resorption-pit assay

Concentration of C-terminal telopeptide of type 1 collagen (CTX-1) in conditioned medium following 14 or 21 days’ culture on slices of human tibia bone was determined with ELISA (Immunodiagnostic System, Inc, Tyne and Wear, United Kingdom.) according to the manufacturer’s protocol. Measurements were performed for osteoclastogenesis assays of three FOA participants expressing OPG-XL and matched healthy controls. Relative activity per osteoclast was calculated by dividing the concentration of CTX-1 by the total number of osteoclasts. Bone resorption was analysed in cell cultures of two FOA participants expressing OPG-XL and matched healthy controls as previously described [28] with Coomassie Brilliant Blue (Sigma-Aldrich, Amsterdam, The Netherlands). Pre-defined areas of each bone slice, covering approximately one-fifth of the total area, were analysed with Image Pro-Plus software (Media Cybernetics, Rockville, MD, USA).

### Histology and immunohistochemistry

Histology was performed as previously described [29]. Overall cellular and tissue structure was visualized with haematoxylin–eosin (H&E) staining. Glycosaminoglycans were visualized by staining with 1% Alcian blue 8-GX (Sigma-Aldrich) and Nuclear Fast Red (Sigma-Aldrich). Calcium deposits were visualized with 2% Alizarin Red S (Sigma-Aldrich). Osteoclasts were stained with tartrate resistant acid phosphatase (TRAcP; Leucocyte acid phosphatase kit, Sigma-Aldrich).

### Gene expression analysis

For osteoclast assays, RNA was extracted from the different cultures at day 7, 14 and 21. For each individual RNA isolation of neo-cartilage and neo-bone, we pooled two organoids for either OPG-XL or CRISPR/Cas9 repaired (wild type, WT) from several independent rounds of differentiations. This generated for neo-cartilage of OPG-XL a total of 5–7 datapoints and for WT a total of 9–12 datapoints in the gene expression plots. For neo-bone, this generated for OPG-XL and WT a total of respectively 10–16 and 7–14 datapoints. Further details and primer sequences can be found in Supplementary Materials and methods and Supplementary Table S5, available at *Rheumatology* online.

### Statistical analysis

Generalized estimating equations (GEE) [30] as implemented in IBM SPSS 25.0 software (IBM Corp., Armonk, NY, USA) was applied to analyse association between phenotype and genotype. GEE methodology provides a method of analysing correlated data that otherwise could not be modelled in a generalized linear model. By applying this method, we were able to effectively adjust for familial dependencies of included participants to obtain β, s.e. and *P*-values for gene expression differences across neo-cartilage and neo-bone [2].

Differences between osteoclast categories defined by the number of nuclei and gene expression were tested using two-way ANOVA and Šidák’s multiple comparison test (GraphPad Prism 6.0 software, GraphPad Software Inc., La Jolla, CA, USA). *P-*values <0.05 were considered statistically significant.

## Results

### Carriers of OPG-XL are characterized by severe OA and osteopenia

*TNFRSF11B* was genotyped in study participants, identifying seven carriers and six non-carriers of OPG-XL among 13 members of a family with early-onset OA (FOA; Fig. 1; Supplementary Table S1, available at *Rheumatology* online). Whole body DEXA scans showed that BMD of non-carriers was similar to that of the general population (Fig. 1C; Supplementary Table S2A, available at *Rheumatology* online). In contrast, carriers of OPG-XL had significantly lower BMD specifically of the femoral neck, narrow neck and total hips, confirming incidence of osteopenia (Fig. 1C; Supplementary Table S2B, available at *Rheumatology* online). No significant difference, however, was observed for lumbar spine BMD. Furthermore, OA features were scored for FOA members based on the semi-quantitative MRI OA knee score (MOAKS). This showed that severe osteophytosis, bone marrow lesions (BML) and cysts have significantly higher prevalence in carriers of the mutation than in non-carriers (Supplementary Table S3, available at *Rheumatology* online). Analysis of the knee radiographs confirmed presence of chondrocalcinosis in three participants expressing OPG-XL. Altogether, this demonstrated that the CCAL1 phenotype in the FOA family is characterized by low BMD and severe cartilage loss, osteophyte formation, and presence of cysts and BML. High mineralization of cartilage was also observed in knee joint of a carrier of the mutation undergoing joint replacement surgery (Fig. 1D, specific regions with calcified cartilage indicated with dashed line).

### Primary chondrocytes with OPG-XL deposit hypertrophic neo-cartilage with low glycosaminoglycan content

We first examined, in an established 3D *in vitro* chondrogenesis model of human primary chondrocytes [29] of a carrier of the OPG-XL mutation (*N* = 1 patient, *n* = 8 replicates), formation of neo-cartilage in comparison with primary chondrocytes from participants of the RAAK study undergoing joint replacement surgery (*N* = 5 patients, *n* = 1 replicate). As shown in Fig. 1E, Alcian blue staining in neo-cartilage with OPG-XL appeared less homogeneous as compared with neo-cartilage deposited by RAAK chondrocytes. Furthermore, H&E staining showed less dense matrix deposition towards the edges of the organoid. Gene expression of common extracellular matrix (ECM) genes and degradation markers highlighted significantly higher expression of *COL1A1*, *COL10A1* and *MMP13* (Fig. 1F; Supplementary Table S6, available at *Rheumatology* online). Moreover, low MGP expression indicated a higher mineralization in the OPG-XL neo-cartilage organoids. Together, these data indicated deposition of low-quality neo-cartilage matrix with a fibrotic (*COL1A1*) and/or hypertrophic (*COL10A1*) and mineralized (*MGP*) phenotype in the presence of OPG-XL.

### Neo-cartilage and neo-bone expressing OPG-XL exhibit altered mineralization and fibrotic phenotype

**Fig. 2:**
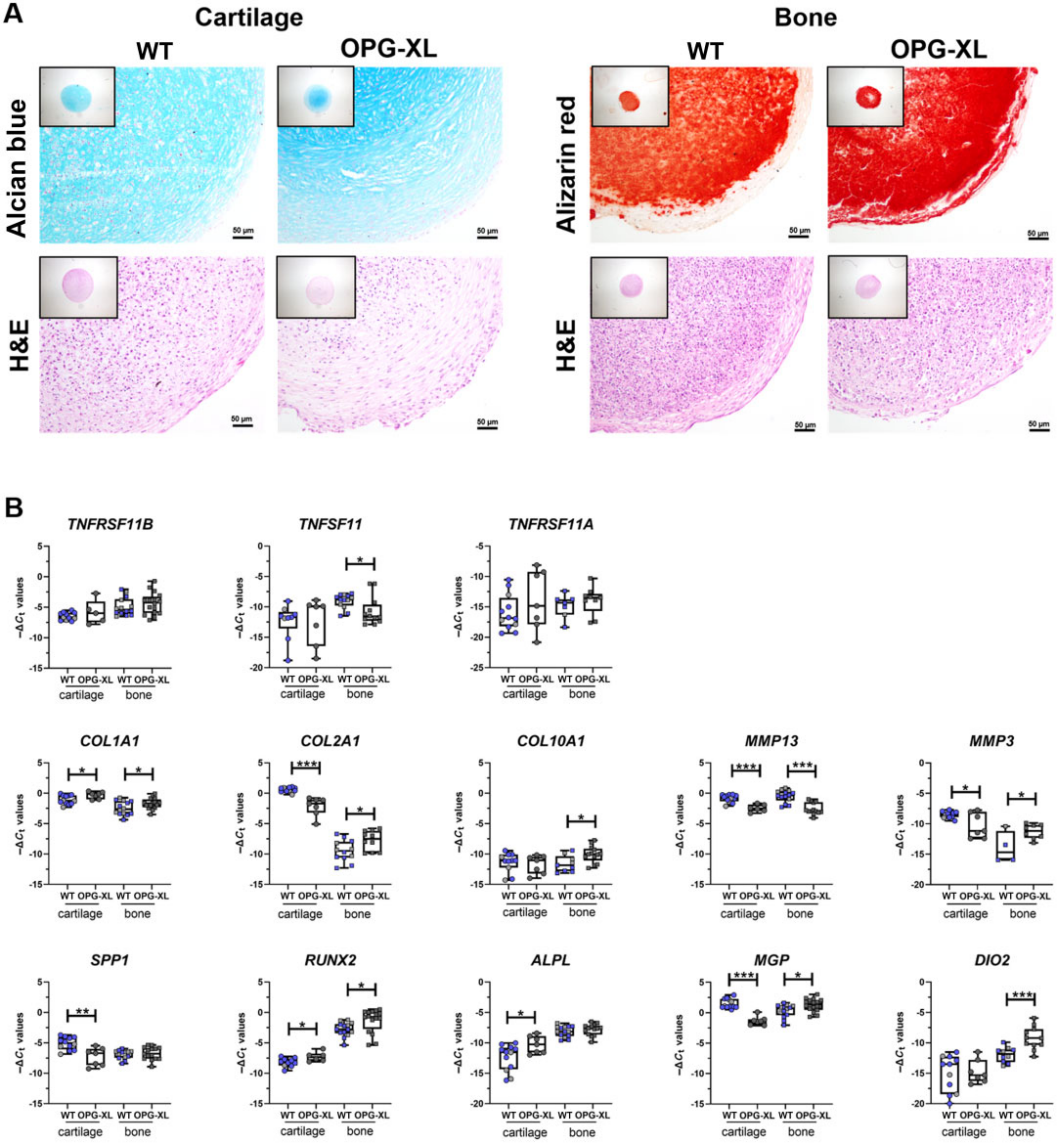
Characterization of OPG-XL neo-cartilage and neo-bone organoids (**A**) Alcian blue, Alizarin red and H&E staining of neo-cartilage and neo-bone. (**B**) Boxplots for −Δ*C*_t_ values of relevant genes for CRISPR/Cas9 control (WT) and OPG-XL organoids following 6 weeks of chondrogenesis with additional 2 weeks of osteogenesis to generate neo-bone (scale bars: 50 µm; neo-cartilage: WT *n* = 9–12 and OPG-XL *n* = 5–7 samples; neo-bone: WT *n* = 7–14 and OPG-XL *n* = 10–16 samples). *P*-values determined with generalized estimation equation while including every independent gene as dependent variable, and mutation status as covariate (*P < 0.05; **P < 10^−4^; ***P < 10^−6^). *ALPL*: alkaline phosphatase; *DIO2*: type 2 deiodinase; H&E: haematoxylin and eosin; *MGP*: matrix Gla protein; *MMP3*: matrix metallopeptidase 3; *MMP13*: matrix metallopeptidase 13; OPG-XL: C-terminal extended osteoprotegerin encoded by *TNFRSF11B* readthrough mutation; *RUNX2*: RUNX family transcription factor 2; *SPP1*: secreted phosphoprotein 1; *TNFSF11*: gene encoding RANK ligand; *TNFRSF11A*: gene encoding RANK; *TNFRSF11B*: gene encoding osteoprotegering or OPG.

To study effects of OPG-XL on deposition of extracellular matrix in the joint, patient hiPSCs carrying the mutation were generated from skin fibroblasts (Supplementary Fig. S1, available at *Rheumatology* online), and both neo-cartilage and neo-bone organoids were created from these hiPSCs and two independent isogenic, CRISPR/Cas9-corrected control (WT) hiPSCs. Structure of deposited neo-cartilage ECM was visualized by histological staining with Alcian blue and H&E (Fig. 2A; Supplementary Fig. S2, available at *Rheumatology* online). This showed ECM deposition across both groups. Matrix, however, was more homogeneous in the two isogenic control organoids as compared with organoids from hiPSCs expressing OPG-XL. Additionally, H&E staining visualized fibrotic ECM particularly towards the outer rim of the OPG-XL neo-cartilage organoids.

In line with the histological observations, quantitative gene expression analysis showed no difference between the two isogenic controls (Fig. 2B, grey- and purple-filled circles in WT boxes). Therefore, samples were analysed together in comparison with OPG-XL samples, which revealed no significant differences in expression levels of *TNFSF11* encoding RANKL, *TNFRSF11A* encoding RANK, or in expression levels of *TNFRSF11B* in neo-cartilage in the presence of OPG-XL. Fibrotic character of neo-cartilage deposited in presence of OPG-XL was confirmed by significantly higher expression of *COL1A1* and lower expression of the cartilage specific *COL2A1* and aggrecan (*ACAN*). Concurrently, strong upregulation of alkaline phosphatase (*ALPL*) and osteoblast characteristic RUNX family transcription factor 2 (*RUNX2*) was detected together with significantly lower expression of SRY-box transcription factor 9 (*SOX9*). Notably, lower expression of *MGP* was highly significant, which was also observed in neo-cartilage deposited by primary chondrocytes with OPG-XL (Fig. 1F). These data indicated a strong shift in cartilage metabolism towards mineralization and transdifferentiation towards osteoblasts. This was in contrast with lower expression levels of secreted phosphoprotein 1 (*SPP1*) and matrix metallopeptidase 13 (*MMP13*).

Structure of deposited neo-bone ECM was visualized by histological staining with Alizarin red and H&E (Fig. 2A; Supplementary Fig. S2, available at *Rheumatology* online). Alizarin red staining was more intense and more homogeneously distributed in the OPG-XL organoids compared with isogenic controls. In the control group, H&E showed a higher concentration of cells within the core of the organoid and an outer rim characterized by fibrosis.

In line with the findings in cartilage organoids, quantitative gene expression analysis of neo-bone showed no significant differences in levels of *TNFRSF11B* and *TNFRSF11A* expression. Unlike neo-cartilage, neo-bone carrying the OPG-XL mutation did show lower expression of *TNFSF11* (Fig. 2B; Table 1; Supplementary Fig. S3, available at *Rheumatology* online). Moreover, neo-bone expressing OPG-XL had significantly higher expression of *COL1A1* and *COL10A1*, but particularly of *DIO2*. Altogether, the results indicate that both neo-cartilage and neo-bone ECM in the presence of OPG-XL is characterized by increased fibrosis and strong mineralization, respectively, indicating a modulatory role for OPG in cartilage and bone formation.

**Table 1:** Gene expression analyses of neo-cartilage and neo-bone organoids

Extracellular matrix					Mineralization				
Gene	FD	β	s.e.	*P*-value^a^	Gene	FD	β	s.e.	*P*-value^a^
Neo-cartilage					Neo-cartilage				
* COL1A1*	**1.7**	**0.8**	**0.3**	**7.4** × **10^−3^**	* SPP1*	**−4.6**	−**2.2**	**0.6**	**2.0** × **10^−4^**
* COL2A1*	**−6.7**	**−2.8**	**0.5**	**3.9** × **10^−7^**	* RUNX2*	**1.9**	**0.9**	**0.4**	**1.0** × **10^−2^**
* COL10A1*	−1.2	−0.3	0.7	6.4 × 10^−1^	* ALPL*	**4.4**	**2.1**	**0.8**	**5.9** × **10^−3^**
* MMP13*	**−2.4**	**−1.6**	**0.3**	**1.4** × **10^−8^**	* MGP*	**−7.1**	−**2.8**	**0.3**	**1.6** × **10^−17^**
* MMP3*	**−3.4**	**−1.8**	**0.7**	**1.5** × **10^−2^**	* POSTN*	1.1	0.2	0.3	5.2 × 10^−1^
* TNFRSF11B*	1.6	0.7	0.8	4.0 × 10^−1^	* ASPN*	1.0	0.0	0.3	9.5 × 10^−1^
* TNFRSF11A*	1.8	2.0	1.9	2.9 × 10^−1^	* DIO2*	1.2	0.3	1.1	8.0 × 10^−1^
* TNFRSF11*	1.0	0.1	1.6	9.7 × 10^−1^	* ANKH*	−1.2	−0.2	0.5	6.9 × 10^−1^
* ACAN1*	**−3.9**	**−2.0**	**0.8**	**1.5** × **10^−2^**					
* SOX9*	**−2.0**	**−1.0**	**0.3**	**4.5** × **10^−3^**					
* COMP*	−1.2	−0.3	0.2	1.8 × 10^−1^					
* ADAMTS5*	1.6	0.6	1.1	5.6 × 10^−1^					
* SMAD3*	**−2.7**	**−1.4**	**0.4**	**4.0** × **10^−4^**					
Neo-bone					Neo-bone				
* COL1A1*	**1.7**	**0.7**	**0.4**	**3.9** × **10^−2^**	* SPP1*	1.1	0.2	0.3	5.8 × 10^−1^
* COL2A1*	**3.3**	**1.7**	**0.7**	**1.2** × **10^−2^**	* RUNX2*	**2.8**	**1.5**	**0.6**	**7.7** × **10^−3^**
* COL10A1*	**4.8**	**1.5**	**0.6**	**5.6** × **10^−3^**	* ALPL*	1.2	0.3	0.3	3.4 × 10^−1^
* MMP13*	**−3.3**	**−2.1**	**0.4**	**7.8** × **10^−9^**	* MGP*	**1.9**	**0.9**	**0.4**	**1.2** × **10^−2^**
* MMP3*	**−14.9**	**2.6**	**1.2**	**2.8** × **10^−2^**	* POSTN*	−1.4	−0.5	0.5	3.14 × 10^−1^
* TNFRSF11B*	1.5	0.6	0.6	3.3 × 10^−1^	* ASPN*	−**3.6**	−**1.8**	**0.5**	**1.4** × **10^−4^**
* TNFRSF11A*	1.8	0.9	1.0	3.8 × 10^−1^	* DIO2*	**6.6**	**2.7**	**0.7**	**3.5** × **10^−5^**
* TNFSF11*	**−3.2**	**−1.7**	**0.7**	**2.0** × **10^−2^**	* IL11*	1.6	0.7	0.5	1.8 × 10^−1^
					* SOST*	**2.9**	**1.5**	**0.7**	**3.5** × **10^−2^**

a Results presented are the average of seven samples for neo-cartilage and 16 samples for neo-bone in OPG-XL carriers compared with 12 samples for neo-cartilage and 14 samples for neo-bone in CRISPR/Cas9-corrected controls (WT) at week 6 following chondrogenesis and additional 2 weeks following osteogenesis. *P*-values determined with generalized estimation equation while including every independent gene as dependent variable, and mutation status as covariate (*P*-values >0.05 are indicated in bold). FD: fold difference.

### Restrained osteoclastogenesis and large polykaryons of monocytes from FOA patients expressing OPG-XL

**Fig. 3:**
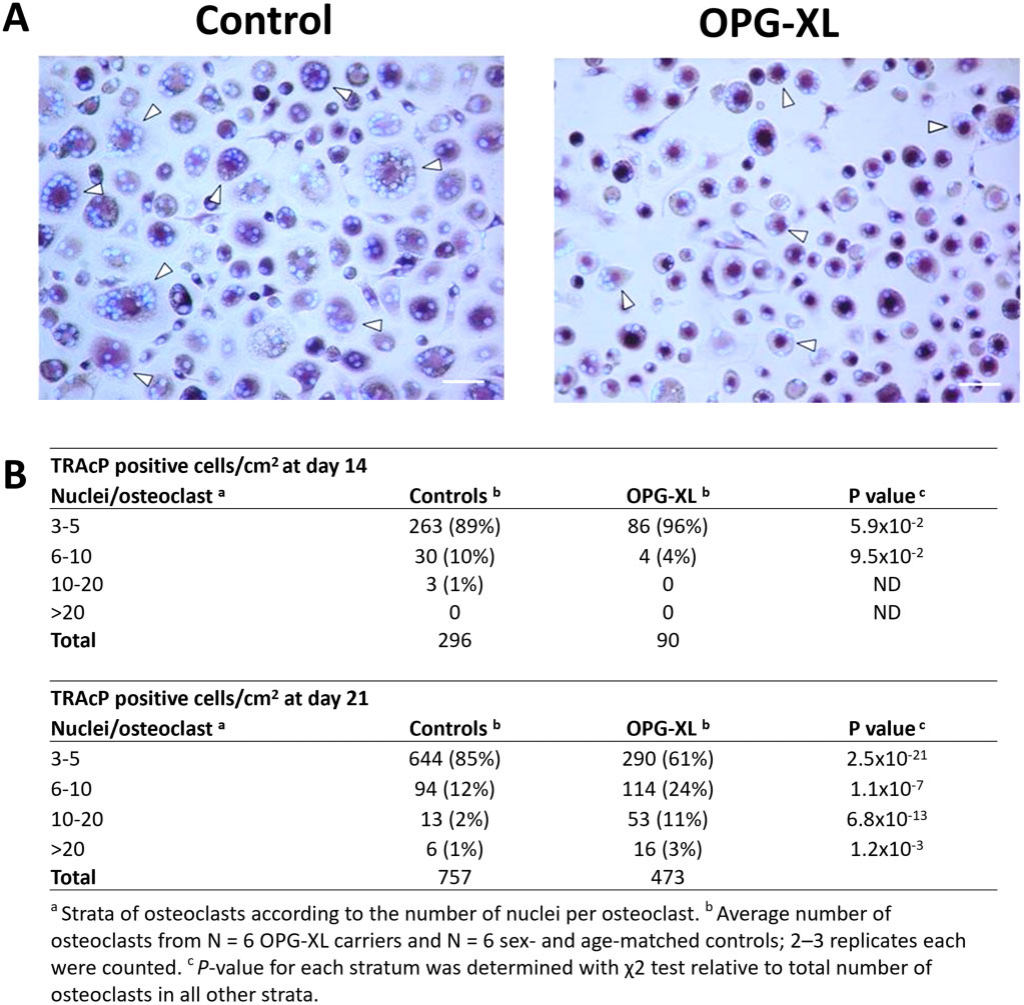
Characterization of osteoclastogenesis (**A**) Representative TRAcP staining of osteoclasts generated from monocytes of OPG-XL carriers and sex- and age-matched controls after 21 days of culture with M-CSF and RANKL (Scale bar = 200 μm). (**B**) Osteoclasts were counted and separated according to the number of nuclei per osteoclast at day 14 and day 21. OPG-XL: C-terminal extended osteoprotegerin encoded by *TNFRSF11B* readthrough mutation; RANKL: RANK ligand; *TRAcP*: Tartrate-resistant acid phosphatase.

Given that OPG plays a key role in osteoclast formation, osteoclastogenesis assays were performed with monocytes from six carriers of OPG-XL mutation and six healthy controls (Fig. 3A). The number of osteoclasts and their respective nuclei was determined at day 14 and day 21 of culture (Supplementary Table S7, available at *Rheumatology* online). As shown in Fig. 3B and Supplementary Table S7, available at *Rheumatology* online, the total number of osteoclasts (cells with >3 nuclei) formed at day 14 in OPG-XL (*n* = 90) is delayed relative to controls (*n* = 296). At day 21, however, we showed a significant accumulation of osteoclasts with high number of nuclei formed in OPG-XL (39% >6 nuclei) compared with controls (15% >6 nuclei; Fig. 3C; Supplementary Table S7, available at *Rheumatology* online). Together, these data indicate that after initial restrained osteoclastogenesis, a relative larger fraction of osteoclasts with large number of nuclei accumulate in the presence of OPG-XL as compared with controls.

### Osteoclasts expressing OPG-XL display similar bone resorptive activity as compared with controls

**Fig. 4:**
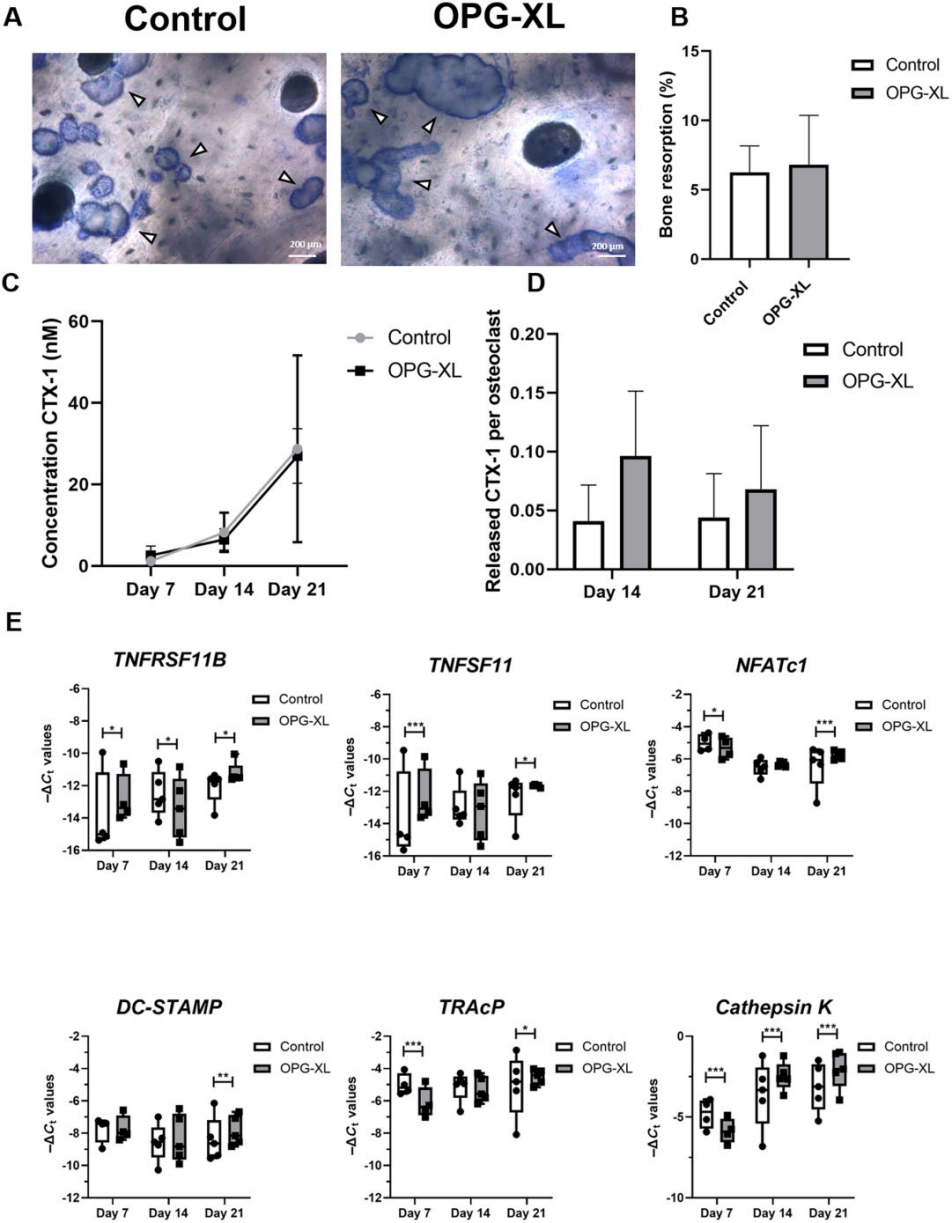
Bone resorption and gene expression for osteoclasts from controls and OPG-XL carriers (**A**) Representative image of bone resorption pits formed by osteoclasts from control and OPG-XL carrier on slices of human tibia bone after 21 days of culture (scale bars: 200 μm). (**B**) Percentage of bone resorption for controls (*N* = 2) and OPG-XL carriers (*N* = 2). (**C**) Concentration of CTX-1 (nM) in conditioned medium after 7, 14 and 21 days of culture (controls and OPG-XL carriers *N* = 3). (**D**) CTX-1 release in conditioned medium per osteoclast at 14 and 21 days of culture (controls *N* = 3 and OPG-XL carriers *N* = 3). (**E**) Boxplots for −Δ*C*_t_ values of genes related to osteoclast formation and activity (*n* = 2–3 replicates from 4–5 controls and OPG-XL carriers). *P*-values determined with two-way ANOVA with Šidák’s multiple comparison analysis (*P < 0.05; **P < 10^−4^; ***P < 10^−6^). CTX-1: C-terminal telopeptide of type 1 collagen; *DC-STAMP*: endrocyte Expressed Seven Transmembrane Protein; *NFATc1*: Nuclear factor of activated T-cells, cytoplasmic 1; OPG-XL: C-terminal extended osteoprotegerin encoded by *TNFRSF11B* readthrough mutation; *TNFSF11*: gene encoding RANK ligand; *TNFRSF11B*: gene encoding osteoprotegerin or OPG; TRAcP: Tartrate-resistant acid phosphatase.

Following morphological characterization of osteoclasts, resorptive activity was assessed. To that end, formation of resorption pits by osteoclasts generated from controls and from carriers of OPG-XL was determined. As shown in Fig. 4, despite lower numbers of osteoclasts in the presence of OPG-XL, overall bone resorption was comparable, with at least equal surface areas of resorption pits after 21 days (Fig. 4A) and amounts of CTX-1 released at days 7, 14 and 21 (Fig. 4B–D). These data indicate that, despite the fact that fewer osteoclasts develop in the presence of OPG-XL, the total bone resorption activity was comparable to controls.

Finally, well-known markers of osteoclast bone resorption activity were analysed by RT-qPCR. As shown in Fig. 4E and Table 2, at day 21 OPG-XL-expressing osteoclasts showed significantly higher levels of *NFATc1* (Nuclear factor of activated T-cells, cytoplasmic 1), *CTSK* (Cathepsin K), *TRAcP* (Tartrate-resistant acid phosphatase) and *DC-STAMP* (Dendrocyte Expressed Seven Transmembrane Protein). This suggests that monocytes carrying the OPG-XL mutation, despite an initial delay in osteoclastogenesis, are prone to differentiate towards osteoclasts with potential for higher bone resorption activity based on gene expression levels.

**Table 2:** Gene expression analyses of osteoclasts from OPG-XL carriers and sex- and age-matched controls

**Marker of osteoclast activity**	FD	*P*-value^a^	Marker of osteoclast activity	FD	*P*-value^a^
*TNFRSF11B*			*DC-STAMP*		
Day 7	2.0	1.7 × 10^−2^	Day 7	1.0	9.9 × 10^−1^
Day 14	−1.9	1.5 × 10^−2^	Day 14	1.2	9.5 × 10^−2^
Day 21	1.8	2.8 × 10^−2^	Day 21	1.5	1.0 × 10^−4^
*TNFSF11*			*TRAcP*		
Day 7	2.4	1.0 × 10^−4^	Day 7	−2.2	1.0 × 10^−4^
Day 14	−1.2	6.4 × 10^−1^	Day 14	−1.1	5.7 × 10^−1^
Day 21	1.6	1.6 × 10^−2^	Day 21	1.5	6.2 × 10^−3^
*NFATc1*			*CTSK*		
Day 7	−1.3	1.2 × 10^−2^	Day 7	−2.1	1.0 × 10^−4^
Day 14	1.1	1.2 × 10^−1^	Day 14	2.2	1.0 × 10^−4^
Day 21	1.6	1.0 × 10^−4^	Day 21	2.1	1.0 × 10^−4^

a Results presented are the average of *n* = 2–3 replicates from *N* = 4–5 OPG-XL carriers compared with age- and sex-matched controls. *P*-values determined with two-way ANOVA with Šidák’s multiple comparison analysis. FD: fold difference; OPG-XL: C-terminal extended osteoprotegerin encoded by *TNFRSF11B* readthrough mutation.

## Discussion

In the current study we explored the mechanism by which OPG-XL causes the characteristic bidirectional phenotype of subchondral bone turnover accompanied by cartilage mineralization in CCAL1 patients. Notably, OPG-XL, displaying 19 additional amino acids at the C-terminal end, was previously found to hamper the formation of a stable HS–OPG–RANKL complex on the osteoblast membrane [17], permitting RANKL mediated osteoclastogenesis in a murine model [11]. Here we show that human osteoclastogenesis with monocytes from OPG-XL carriers relative to sex- and age-matched controls, after an initial delay, indeed have enhanced osteoclastogenesis towards prominent large and active osteoclasts. By further characterization of OPG-XL by employing hiPSCs from a carrier of the mutation and two isogenic CRISPR/Cas9-corrected controls in cartilage and bone organoids, we demonstrated for the first time that, likely due to interference with RANKL–HS–OPG, the mutation at the CCAL1 locus directly affects healthy osteoblast and chondrocyte states towards mineralization via respectively DIO2 and MGP functions. The fact that OPG/RANKL as well as DIO2 [29] and MGP [2, 31] are intrinsically involved in joint tissue (patho)physiology might indicate a link to common age-related osteoarthritis.

The hiPSC-derived neo-cartilage tissue deposited by OPG-XL chondrocytes, relative to isogenic controls, revealed a fibrotic histological phenotype with marked downregulation of *COL2A1: COL1A1* expression ratio and most notable, downregulation of *MGP* expression. MGP is a well-known inhibitor of ectopic bone formation [5] and *MGP* is a robust OA risk gene [24, 31] with the risk allele associated with lower gene expression levels [2, 31]. Hence, our data, showing lower expression of *MGP* in OPG-XL neo-cartilage organoids, suggest that OPG-XL directly affects propensity of chondrocytes to enter a mineralized OA state. On a different note, we showed that in OPG-XL neo-cartilage organoids the OPG–RANKL–RANK triad was not changed.

Neo-osseous tissue deposited by osteoblasts from hiPSCs carrying the mutation relative to their isogenic controls did display high calcification as reflected by the prominent Alizarin red staining concurrent with notable high expression of *DIO2. DIO2*, encoding type 2 deiodinase enzyme, essentially facilitates bone formation and mineralization [32]. Together, results from our human OPG-XL cartilage and bone organoids demonstrate that the mutation directly affects chondrocyte and osteoblast gene expression profiles marking matrix mineralization processes. We hypothesize that this is due to the impaired binding of OPG with HS, likely in interaction with RANKL as recently shown [11]. Since the prominent Alizarin red staining of neo-osseous tissue, concurrent with *DIO2* upregulation could explain the extensive phenotypic foci of calcified cartilage observed in affected articular cartilage tissue of CCAL1 family members, we hypothesize that the chondrocalcinosis observed in OPG-XL carriers is likely not preceding OA onset in cartilage but arises merely during ongoing OA pathophysiology, i.e. when chondrocytes have a tendency to undergo trans-differentiation to osteoblasts [33].

By performing osteoclastogenesis assays of OPG-XL carriers relative to age- and sex-matched controls, we showed that, although significantly lower in number, osteoclastogenesis in OPG-XL carriers resulted in high nucleated osteoclasts (Fig. 3). These multi-nuclear osteoclasts appear at least equally active as controls as demonstrated by the comparable resorbed bone surface and released levels of CTX-1 (Fig. 4). The fact that we were able to quantify the resorption pits only for two donors and controls has likely resulted in limited statistical power to detect small differences. This hypothesis could hence explain the bidirectional phenotype of the OPG-XL carriers characterized by higher cartilage mineralization and osteopenia. A similar case was previously reported by Zhang *et al.* [34], where MGP knockout mice have a characteristic phenotype of premature bone mineralization and osteopenia. Since RANKL was found to induce osteoclast fusion whereas OPG blocked fusion of highly mobile osteomorphs [15], it is tempting to link the role of immobilized OPG on the surface of osteoblasts to *in vivo* recycling of osteoclasts [35]. Nevertheless, our study does not allow us to distinguish whether the OPG-XL mutation results in highly nucleated and active osteoclasts due to enhanced RANKL availability or due to dysfunctional blocking of osteomorph fusion.

Among genes significantly changed in the presence of OPG-XL we observed that *MMP13* was higher in the primary chondrocytes while lower in the iPSC-derived chondrocytes. This suggests that *MMP13* itself is not directly related to the changes resulting from OPG-XL, but rather, that the effect results from individual variation and/or differences in the maturity of neo-cartilage derived from hiPSCs as compared with that of primary chondrocytes.

By precise genetic engineering of hiPS cells derived from affected CCAL1 family members, while applying established differentiation protocols towards human biomimetic cartilage and osseous organoids [36], we could study in multiple biological replicates how expression of OPG-XL could result in the characteristic bidirectional phenotype of subchondral bone turnover accompanied by cartilage mineralization. Strength of using isogenic controls is that this allows us to study unbiased effects of the mutation, independent of variation between family members such as genetics, sex or age. As such, we are confident that our approach was able to create reliable data highly translating to the human *in vivo* situation while complying with the societal need to reduce animal studies [37, 38]. A potential drawback of our study is that we were not able to model direct interaction between cells populating cartilage and bone: chondrocytes, osteoblast and osteoclasts. In this context, using the isogenic pairs of our patient-derived hiPSCs in human joint-on-chip models, currently being developed, could further address the molecular mechanism underlying the bi-directional effect of OPG on hard and soft tissues [39, 40].

In conclusion, our data demonstrated that expression of OPG-XL in human cartilage and bone not only enhances RANKL-induced osteoclastogenesis, but also directly affects chondrocyte and osteoblast states towards matrix mineralization via functions of respectively MGP and DIO2. We advocate that the bidirectional phenotype of subchondral bone turnover accompanied by cartilage mineralization is a characteristic process that occurs in CCAL1 patients at early ages but which can be extrapolated to common age-related OA patients. Vice versa, our data suggest that in cartilage, proper binding of OPG to HS on chondrocytes intrinsically contributes to a healthy unmineralized tissue state while in bone it supports the steady state turnover with adaptive activity involving osteoblasts and osteoclasts.

## Supplementary Material

keac232_Supplementary_Data

## Data Availability

The data that support the funding of this study are available from the corresponding author upon request.
