# Supplementary material for: Mutation in the CCAL1 locus accounts for bidirectional process of human subchondral bone turnover and cartilage mineralization
Source: Rheumatology (Oxford). 2022 Apr 12;62(1):360–72. doi: 10.1093/rheumatology/keac232 (PMC9788812; doi:10.1093/rheumatology/keac232)
Supplement: keac232_Supplementary_Data [file keac232_supplementary_data.docx]

**SUPPLEMENTARY MATERIAL**

**‘****Mutation in the CCAL1 locus accounts for bidirectional process of human subchondral bone turnover and cartilage mineralization.’**

**Supplementary Methods and Materials**

***Radiographic analyses***

DEXA scans of 253 controls were used to compare results to the general population. OA features were scored based on semi quantitative MRI OA knee score (MOAKS(1)) highlighting different characteristics (e.g. bone marrow lesions, osteophytes, cysts, and loss of cartilage full thickness) at 14 articular subregions in the knee. Skin fibroblasts were obtained from a OPG-XL mutation carrier to generate hiPSCs, while primary chondrocytes were obtained from another OPG-XL mutation carrier for analysis of chondrogenesis. Six carriers of OPG-XL and six gender and age-matched healthy controls donated blood for osteoclastogenesis assay. Ethical approval for the Research Arthritis and Articular Cartilage (RAAK) study (P08.239 and P19.013(2) and the familial OA (FOA) study (P12-256) was obtained from the medical ethics committee (METC) of the LUMC. Approval for the generation of hiPSCs from skin fibroblasts of healthy donors is available under METC LUMC number P13.080. Informed consent was provided by all donors and participants of the FOA family.

***CRISPR/Cas9 correction of OPG-XL patient hiPSCs***

For CRISPR/Cas9 correction of OPG-XL in patient-derived hiPSCs was performed with two single guide RNAs (sgRNAs). sgRNAs were designed by *in silico* tools (Rgenome.net and MultiCrispr.net) and selected based on predicted highest specificity and least off-target effects: gRNA1 (5’-AAAAATAAGCTGCTTATTACTGG-3’), and gRNA2 (5’-AAGCTGCTTATTA CTGGAAATGG-3’). The sgRNAs were cloned into a CRISPR/Cas9 plasmid carrying sequences for expression of green fluorescent protein (PX458), and co-transfected using Lipofectamine Stem Reagent (Thermo Fisher Scientific Inc) with a single-stranded oligo donor repair template (ssODN 5'-CACTGAAAGCCTCAAGTGCCTGAGAAACAGTTTACTCAT CCATGGGATCTCGCCAATTGTGAGGAAACAGCTCAATGGCGATTTCGAGTTATAAGCAGCTTATTTTTACTGATTGGACCTGGTTACC-3') to achieve homologous directed repair (HDR). Twenty-four hours after transfection of gRNAs and ssODN, single-cell-sorting for green fluorescent protein positive cells was performed with FACSAria-I (BD Biosciences), and hiPSCs were seeded at low density (270 cells/cm^2^) for clonal expansion. After eight days, colonies were collected and reseeded as single-cells in 96-well plates with TESR-E8 and CloneR (STEMCELL Technologies). DNA was obtained from single colonies using Quick Extract (Lucigen). Region of interest was amplified with PCR and screening for homozygous CRISPR/Cas9-corrected colonies (wt) was done by restriction with PsiI (New England Biolabs). Two homozygous clones (B89 and C81 from gRNA2 and gRNA1, respectively) were identified and repair of the readthrough mutation was confirmed by Sanger sequencing (**Supplementary Figure S1D-F**).

***Isolation of blood cells and*** ***osteoclastogenesis***

Peripheral blood mononuclear cells (PBMCs) were isolated from whole blood using Ficoll density gradient centrifugation as previously described.(3) CD14-positive monocytes were collected by negative selection using magnetic MACS microbeads according to the manufacturer’s instructions. Cells were then seeded onto slices of human tibia bone of healthy individuals and cultured in medium composed of α-MEM supplemented with 10% fetal calf serum (HyClone I, Thermo Fisher Scientific Inc) and antibiotics following pre-treatment with 10 ng/ml macrophage colony-stimulating factor (M-CSF, R&D Systems) for three days. Osteoclastogenesis was induced by addition of 2 ng/ml RANKL (R&D Systems) in the presence of 10 ng/ml M-CSF. Cultures were maintained at 37°C and 5% CO2 with medium refreshed twice weekly.

***Gene expression analysis***

RNA was isolated and total mRNA (150 ng) was processed with first strand cDNA kit according to manufacturer’s protocol (Roche Applied Science). Genes of interest were determined by preamplification with TaqMan preamp master mix (Thermo Fisher Scientific Inc) and subsequent RT-qPCR in triplicate with BiomarkTM 96.96 Dynamic Arrays (Fluidigm) and integrated fluidic circuit (IFC) chip. Quality control of the data was performed as previously described.(4) Unsuccessful differentiation experiments defined by the minimum detected expression of *COL2A1* for human primary chondrocytes and BMSCs neo-cartilage, were disregarded. Relative gene expression (−ΔCt-values) was calculated using levels of glyceraldehyde 3-phosphate dehydrogenase (*GAPDH*) and acidic ribosomal phosphoprotein P0 (*ARP*) as housekeeping genes. The RT-qPCR primers are listed in **Supplementary Table S5**.

***Histology and immunohistochemistry***

Histology was performed as previously described.(4) Overall cellular and tissue structure was visualized with hematoxylin-eosin (H&E) staining. Glycosaminoglycans were visualized by staining with 1% Alcian Blue 8-GX (Sigma-Aldrich) and Nuclear Fast red (Sigma-Aldrich). Calcium deposits were visualized with 2% Alizarin Red S (Sigma-Aldrich).

Formation of osteoclasts was assessed after 14 and 21 days of culture on plastic. Osteoclasts were fixed in 4% formaldehyde for 10 min at ambient temperature and stained for tartrate resistant acid phosphatase (TRAcP) using a commercial kit (Leukocyte acid phosphatase kit, Sigma–Aldrich) according to the manufacturer’s instructions. Nuclei were visualized with 4’6-diamidino-2-phenylindole dihydrochloride (DAPI, Sigma-Aldrich). Only cells with three or more nuclei were considered osteoclasts. Results are presented as osteoclasts per cm^2^ or in nuclei per osteoclast.

**REFERENCES**

1. Hunter DJ, Guermazi A, Lo GH, Grainger AJ, Conaghan PG, Boudreau RM, et al. Evolution of semi-quantitative whole joint assessment of knee OA: MOAKS (MRI Osteoarthritis Knee Score). Osteoarthritis Cartilage. 2011;19(8):990-1002.

2. Ramos YF, den Hollander W, Bovee JV, Bomer N, van der Breggen R, Lakenberg N, et al. Genes Involved in the Osteoarthritis Process Identified through Genome Wide Expression Analysis in Articular Cartilage; the RAAK Study. PLoS One. 2014;9(7):e103056.

3. Sprangers S, Schoenmaker T, Cao Y, Everts V, de Vries TJ. Different Blood-Borne Human Osteoclast Precursors Respond in Distinct Ways to IL-17A. J Cell Physiol. 2016;231(6):1249-60.

4. Bomer N, den Hollander W, Ramos YF, Bos SD, van der Breggen R, Lakenberg N, et al. Underlying molecular mechanisms of DIO2 susceptibility in symptomatic osteoarthritis. Ann Rheum Dis. 2015;74(8):1571-9.

**Supplementary Figure S1. Characterization of generated OPG-XL hiPSCs and CRISPR/Cas9-corrected clonal screening.** (**A**) Bright field microscopy image of a representative OPG-XL mutated hiPSC colony. (**B**) Immunofluorescent staining for NANOG, SSEA-4, and OCT3/4 confirming pluripotency. (**C**) Expression of β3-Tubulin, CD31, and AFP upon spontaneous differentiation into the three different lineages (ectoderm, mesoderm and endoderm, respectively). Nuclei are stained with Dapi (blue). (**D**) Clonal screening for OPG-XL repaired mutation by genomic PCR followed by PsiI digestion. Patient DNA shows three bands after PsiI digestion, whereas repaired clones B89 (gRNA1) and C81 (gRNA2) together with the positive control show two bands. (**E**) Sanger sequencing and (**F**) karyotyping of 2 corrected clones and OPG-XL hiPSC line.


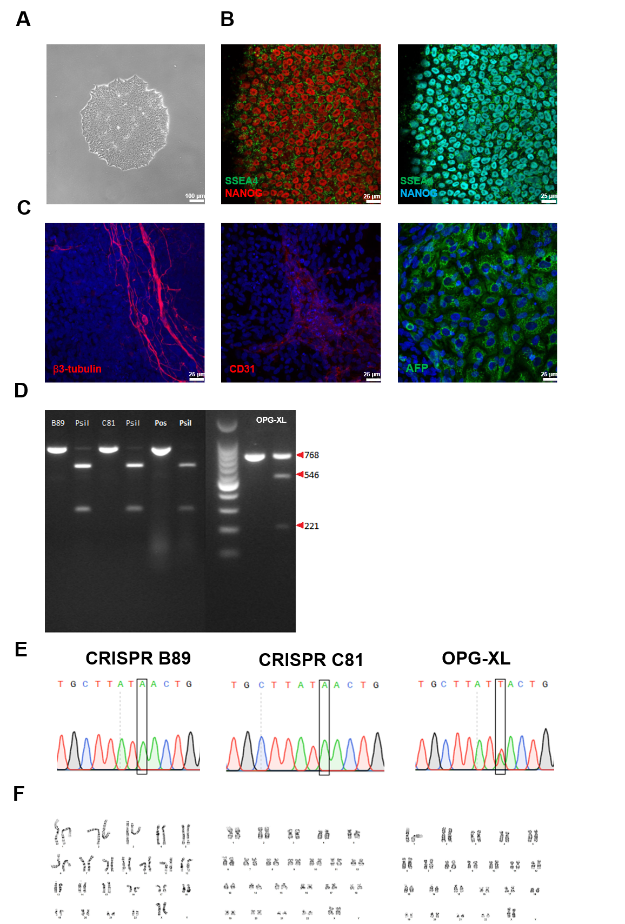


**Supplementary Figure S2. Alcian Blue and Alizarin red staining of different neo-cartilage and neo-bone organoids.** Histology of organoids resulting from different experiments at day 42 of chondrogenesis stained with Alcian Blue (n=2 independent differentiations) and day 14 of osteogenesis (n=4 independent differentiations) stained with Alizarin red (scale bars: 100µm).


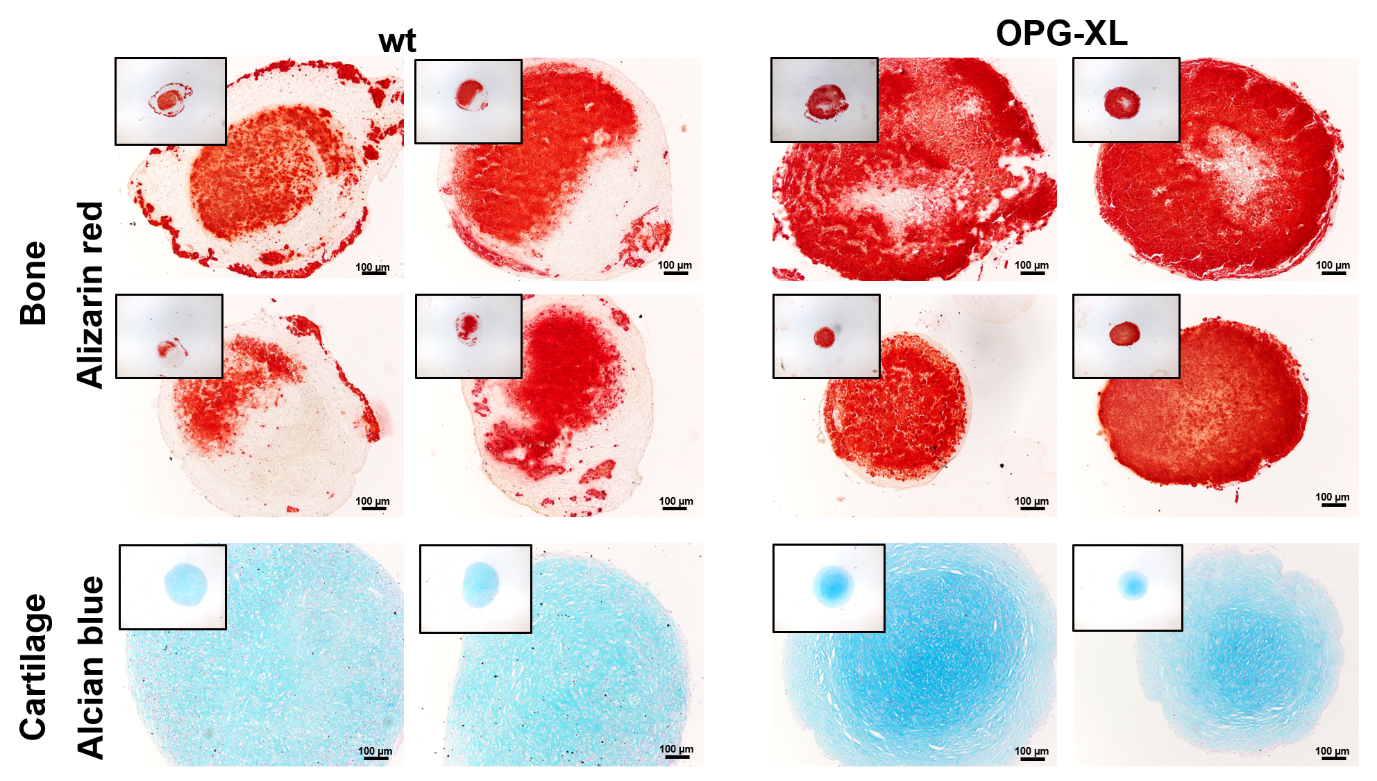


**Supplementary Figure S3. Ratios of *TNFSF11/TNFRSF11A*, *TNFRSF11B/TNFSF11* and *COL2A1/COL1A1* in neo-cartilage and neo-bone of the OPG-XL mutation and CRISPR/Cas9-corrected samples.** Results shown are at day 42 of chondrogenesis and day 14 of osteogenesis (neo-cartilage: n=9-12 for CRISPR-corrected control and n=5-7 for OPG-XL samples; neo-bone: n=7-14 for CRISPR-corrected control and n=10-16 for OPG-XL samples.; *P-value<0.05; **P-value<10^-4^; ***P-value<10^-6^)


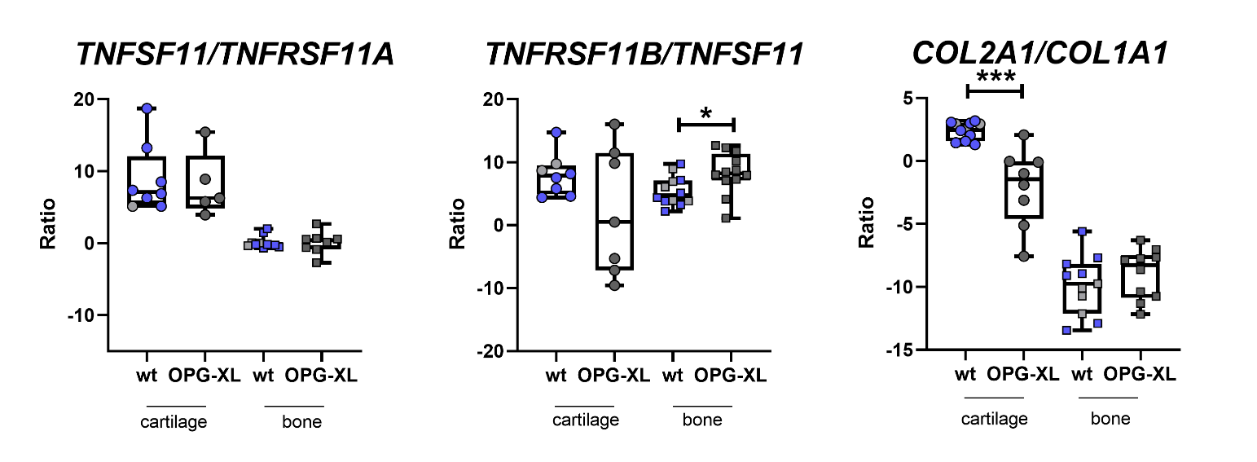


**Supplementary Table S1. Characteristics of FOA participants.**

|  | **OPG-XL non-carrier** | **OPG-XL carriers** |
| --- | --- | --- |
| **Sex (%)** |  |  |
| Male | 66.7 | 42.9 |
| Female | 33.3 | 57.1 |
| **Age (years)** |  |  |
| Minimum | 23.1 | 32.5 |
| Maximum | 60.5 | 61.6 |
| Average | 41.7 ± 14.3 | 51.7 ± 12.3 |
| **BMI (Kg/m^2^)** |  |  |
| Minimum | 22.3 | 24.9 |
| Maximum | 29.1 | 30.1 |
| Average | 24.8 ± 3 | 27.0 ± 1.9 |

OPG-XL: high impact read-through mutation in *TNFRSF11B* at CCAL1 locus.

**Supplementary Table S2. Skeletal characteristics of participants determined with DEXA scans at different regions of the skeleton.**

**A**

| **DEXA** | **FOA non-carriers** | **Healthy controls** |
| --- | --- | --- |
| **BMD (g/cm2)** |  |  |
| Skull | 2.5 ± 0.3 | 2.5 ± 0.3 |
| Femoral neck | 0.9 ± 0.1 | 0.9 ± 0.1 |
| Narrow neck | 1.1 ± 0.2 | 1.1 ± 0.1 |
| Lumbar spine | 1.1 ± 0.2 | - |
| Total body | 1.1 ± 0.2 | 1.2 ± 0.1 |
| Total hips | 1.0 ± 0.1 | 1.0 ± 0.1 |
| **Narrow neck width (cm)** | 3.6 ± 0.4 | 3.5 ± 0.4 |

Results presented are the average of FOA non-carriers (N=7) versus non-related healthy controls (N=253).

**B**

| DEXA | FOA non-carrier | FOA mutation | P value ^a^ |
| --- | --- | --- | --- |
| BMD (g/cm^2^) |  |  |  |
| Skull | 2.5 ± 0.3 | 2.3 ± 0.5 | 4.3x10^-1^ |
| Femoral neck | 0.9 ± 0.1 | 0.7 ± 0.1 | **1.8x10^-2^** |
| Narrow neck | 1.1 ± 0.2 | 0.8 ± 0.1 | **4.5x10^-3^** |
| Lumbar spine | 1.1 ± 0.2 | 1.1 ± 0.2 | 8.8x10^-1^ |
| Total body | 1.1 ± 0.2 | 1.2 ± 0.2 | 1.8x10^-1^ |
| Total hips | 1.0 ± 0.1 | 0.8 ± 0.1 | **7.2x10^-3^** |
| Narrow neck width (cm) | 3.6 ± 0.4 | 3.9 ± 0.2 | **4.1x10^-4^** |
| T-score |  |  |  |
| Hips | -0.1 ± 1.4 | -1.4 ± 0.6 | **1.2x10^-2^** |
| Lumbar spine | -0.1 ± 1.5 | 0.2 ± 1.8 | 8.8x10^-1^ |
| MOAKS |  |  |  |
| BML and Cysts (#) | 0.3 ± 0.5 | 18.6 ± 14.1 | **2.4x10^-5^** |

Results presented are the average of FOA members (7 carriers versus 6 non-carriers of the OPG-XL mutation; # number).

^a^P-value was determined by performing a generalized estimation equation (GEE), with BMD, Narrow neck, T-score, and BML and cysts as dependent variable, and age, sex and BMI as covariate.

OPG-XL: high impact readthrough OPG mutation at CCAL1 locus.

**Supplementary Table S3. MOAKS grading of knee joints.**

| **MOAKS** | **Grades** | | | |
| --- | --- | --- | --- | --- |
| **Osteophytes (Size)** | **0** | **1** | **2** | **3** |
| OPG-XL non-carrier (%) | 95.8 ± 5.3 | 4.2 ± 5.3 | 0 | 0 |
| OPG-XL carrier (%) | 30.0 ± 23.3 | 31.7 ± 13.4 | 21.7 ± 9.0 | 16.7 ± 17.7 |
| P value ^a^ | **6.8x10^-4^** | **2.3x10^-4^** | **3.4x10^-4^** | **1.7x10^-3^** |
| **BML & Cyst (Size)** | **0** | **1** | **2** | **3** |
| OPG-XL non-carrier (%) | 98.9 ± 1.7 | 1.1 ± 1.7 | 0 | 0 |
| OPG-XL carrier (%) | 57.3 ± 19.6 | 30.0 ± 7.1 | 5.3 ± 6.1 | 7.3 ± 8.3 |
| P value ^a^ | **1.7x10^-10^** | **1.0x10^-30^** | **2.0x10^-2^** | **2.4x10^-2^** |
| **BML & Cyst (%)** | **0** | **1** | **2** | **3** |
| OPG-XL non-carrier (%) | 99.4 ± 1.4 | 0 | 0 | 0.6 ± 1.4 |
| OPG-XL carrier (%) | 63.3 ± 17.0 | 4.7 ± 6.5 | 4.7 ± 7.3 | 26.0 ± 7.6 |
| P value ^a^ | **2.0x10^-9^** | 1.0x10^-1^ | **7.3x10^-2^** | **0.0x10^-30^** |
| **Cartilage (%)** | **0** | **1** | **2** | **3** |
| OPG-XL non-carrier (%) | 91.8 ± 8.1 | 5.8 ± 5.8 | 3.0 ± 3.6 | 0.9 ± 1.5 |
| OPG-XL carrier (%) | 56.4 ± 19.3 | 7.6 ± 4.3 | 4.4 ± 2.1 | 33.1 ± 17.3 |
| P value ^a^ | **3.7x10^-4^** | 7.8x10^-1^ | 3.6x10^-1^ | **4.5x10^-10^** |

Results presented are the average of FOA members (5 carriers versus 6 non-carriers of the OPG-XL mutation; 2 carriers were excluded due to knee prosthetics).

^a^P-value was determined by performing a generalized estimation equation (GEE), with osteophyte (size), BML and cysts size and percentage, and cartilage percentage as dependent variable, and age, sex and BMI as covariate.

OPG-XL: high impact readthrough OPG mutation at CCAL1 locus.

**Supplementary Table S4. Osteoclastogenesis assay of OPG-XL family members against age- and sex-matched controls.**

| **Pair** | **Participant** | **Sex** | **Age** |  |
| --- | --- | --- | --- | --- |
| 1 | Control | Male | 49 |  |
|  | OPG-XL | Male | 39 |  |
| 2 | Control | Female | 53 |  |
|  | OPG-XL | Female | 62 |  |
| 3 | Control | Female | 60 |  |
|  | OPG-XL | Female | 60 |  |
| 4 | Control | Male | 65 |  |
|  | OPG-XL | Male | 48 |  |
| 5 | Control | Male | 54 |  |
|  | OPG-XL | Male | 59 |  |
| 6 | Control | Female | 57 |  |
|  | OPG-XL | Female | 61 |  |

FOA: individual with familial early-onset osteoarthritis, carrier of high impact readthrough mutation in *TNFRSF11B* at CCAL1 locus.

**Supplementary Table S5. Matrix, mineralization, osteoclastogenesis and housekeeping primer sequences.**

|  |  | |
| --- | --- | --- |
| **Matrix Genes** | **Fwd** | **Rvs** |
| *ADAMTS5* | 5'-CGTGTACTTGGGCGATGACA-3' | 5'-CTGTTGTTGCACACCCCTCT-3' |
| *ACAN1* | 5'AGAGACTCACACAGTCGAAACAGC-3' | 5'-CTATGTTACAGTGCTCGCCAGTG-3' |
| *COL10A1* | 5'-GGCAACAGCATTATGACCCA-3' | 5'-TGAGATCGATGATGGCACTCC-3' |
| *COL1A1* | 5'-GTGCTAAAGGTGCCAATGGT-3' | 5'-ACCAGGTTCACCGCTGTTAC -3' |
| *COL2A1* | 5'-CTACCCCAATCCAGCAAACGT-3' | 5'-AGGTGATGTTCTGGGAGCCTT-3' |
| *COMP* | 5'-ACAATGACGGAGTCCCTGAC-3' | 5'-TCTGCATCAAAGTCGTCCTG-3' |
| *MMP13* | 5'-TTGAGCTGGACTCATTGTCG-3' | 5'-GGAGCCTCTCAGTCATGGAG-3' |
| *SOX9* | 5'-CCCCAACAGATCGCCTACAG-3' | 5'-CTGGAGTTCTGGTGGTCGGT-3' |
| SMAD3 | 5'-GCCCCTTTCAGGTAACCGTC-3' | 5'-GAAGCGGCTGATGCTCCTTA-3' |
| *MMP3* | 5'-GAGGCATCCACACCCTAGGTT-3' | 5'-TCAGAAATGGCTGCATCGATT-3' |
| **Mineralization genes** | **Fwd** | **Rvs** |
| *ALPL* | 5'-CAAAGGCTTCTTCTTGCTGGTG-3' | 5'-CCTGCTTGGCTTTTCCTTCA-3' |
| *ASPN* | 5'-ACACGTTTTGGAAATGAGTGC-3' | 5'-GAACACCGTCACCCCTTCAA-3' |
| *MGP* | 5'-CGCCCCCAGATTGATAAGTA-3' | 5'-TCTCCTTTGACCCTCACTGC-3' |
| *POSTN* | 5'-TACACTTTGCTGGCACCTGT-3' | 5'-TTTAAGGAGGCGCTGATCCA-3' |
| *RUNX2* | 5'-CAATTTCCTCCTTGCCCCTCA-3' | 5'-TCGGATCTACGGGAATACGCA-3' |
| *SPP1* | 5'-GCCAGTTGCAGCCTTCTCA-3' | 5'-AAAAGCAAATCACTGCAATTCTCA-3' |
| *TNFRSF11A* | 5'-GAAGCTCAGCCTTTTGCTCA-3' | 5'-GGGAACCAGATGGGATGTCG-3' |
| *TNFRSF11B* | 5'-TTGATGGAAAGCTTACCGGGA-3' | 5'-TCTGGTCACTGGGTTTGCATG-3' |
| *TNFSF11* | 5'-CAACAAGGACACAGTGTGCAA-3' | 5'-AGGTACAGTTGGTCCAGGGT-3' |
| *DIO2* | 5'-TTCCAGTGTGGTGCATGTCTC-3' | 5'-AGTCAAGAAGGTGGCATGTGG-3' |
| *IL11* | 5'-CTCTACAGCTCCCAGGTGTGC-3' | 5'-AGGTAGGACAGTAGGTCCGCT-3' |
| *SOST1* | 5'-GAGCTGGAGAACAACAAGACCA-3' | 5'-AGCTGTACTCGGACACGTCTTTG-3' |
| **Osteoclastogenesis genes** | **Fwd** | **Rvs** |
| *NFATc1* | 5'-AGCAGAGCACGGACAGCTATC-3' | 5'-GGTCAGTTTTCGCTTCCATCTC-3' |
| *DC-STAMP* | 5'-ATTTTCTCAGTGAGCAAGCAGTTTC-3' | 5'-AGAATCATGGATAATATCTTGAGTTCCTT-3' |
| *TRAcP* | 5'-CACAATCTGCAGTACCTGCAAGAT-3' | 5'-CCCATAGTGGAAGCGCAGATA-3' |
| *Cathepsin K* | 5'-CCATATGTGGGACAGGAAGAGAGTT-3' | 5'-TGCATCAATGGCCACAGAGA-3' |
| **Housekeeping genes** | **Fwd** | **Rvs** |
| *GAPDH* | 5'-TGCCATGTAGACCCCTTGAAG-3' | 5'-ATGGTACATGACAAGGTGCGG-3' |
| *ARP* | 5'-CACCATTGAAATCCTGAGTGATGT-3' | 5'-TGACCAGCCGAAAGGAGAAG-3' |

**Supplementary Table S6. Gene expression analyses of primary chondrocytes**

| **Extracellular matrix genes** | | | | |
| --- | --- | --- | --- | --- |
| **Genes** | **Fold change** | **Beta** | **SE** | **P value ^a^** |
| ***COL1A1*** | **13.8** | **3.8** | **0.3** | **1.0x10^-30^** |
| *COL2A1* | -1.8 | -0.8 | 0.6 | 1.3x10^-1^ |
| ***COL10A1*** | **6.9** | **2.8** | **0.3** | **8.6x10^-16^** |
| *ACAN* | 1.2 | 0.3 | 0.4 | 4.7x10^-1^ |
| *SOX9* | 2.4 | 1.2 | 0.7 | 7.8x10^-2^ |
| *ADAMTS5* | -1.5 | -0.6 | 1.0 | 5.4x10^-1^ |
| ***MMP13*** | **5.6** | **-2.5** | **0.6** | **1.0x10^-5^** |

Results presented are the average of one carrier of the OPG-XL mutation (8 replicates) versus five independent OA patients that underwent joint replacement surgery following three weeks of chondrogenesis.

^a^P-value was determined by performing a generalized estimation equation (GEE) of -delta CT values, with every independent gene as a dependent variable, and mutation condition as covariate.

**Supplementary Table S7. Number of osteoclasts (>3 nuclei) formed in osteoclastogenesis.**


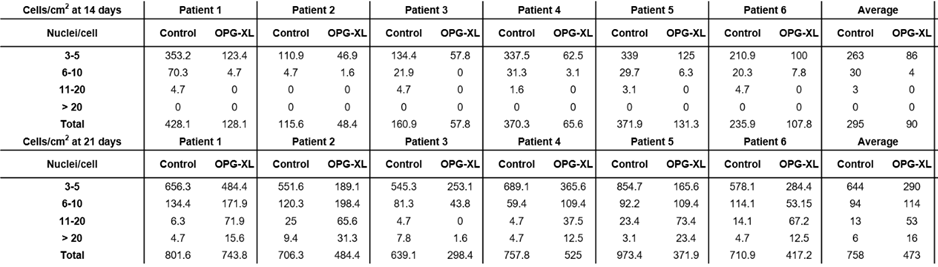


Results are presented for each participant (FOA members expressing OPG-XL and age- and sex-matched controls) stratified for number of nuclei per osteoclast and counted in triplicate at day 14 and at day 21.

OPG-XL: high impact readthrough mutation in *TNFRSF11B* at CCAL1 locus.
